# On the Generalization of Lehmer Problem and High-Dimension Kloosterman Sums

**DOI:** 10.1155/2014/726053

**Published:** 2014-07-16

**Authors:** Guohui Chen, Han Zhang

**Affiliations:** ^1^College of Mathematics and Statistics, Hainan Normal University, Hainan 571158, China; ^2^School of Mathematics, Northwest University, Xi'an, Shaanxi 710127, China

## Abstract

For any fixed integer *k* ≥ 2 and integer *r* with (*r*, *p*) = 1, it is clear that there exist *k* integers 1 ≤ *a*_*i*_ ≤ *p* − 1 (*i* = 1, 2, …, *k*) such that *a*_1_ *a*_2_ ⋯ *a*_*k*_ ≡ *r* mod *p*. Let *N*(*k*, *r*; *p*) denote the number of all (*a*_1_, *a*_2_, ⋯ *a*_*k*_) such that *a*_1_ *a*_2_ ⋯ *a*_*k*_ ≡ *r* mod *p* and 2†(*a*_1_ + *a*_2_ + ⋯ + *a*_*k*_). In this paper, we will use the analytic method and the estimate for high-dimension Kloosterman sums to study the asymptotic properties of *N*(*k*, *r*; *p*) and give two interesting asymptotic formulae for it.

## 1. Introduction

Let *p* be an odd number. For each integer *a* with 1 ≤ *a* ≤ *p* − 1, it is clear that there exists one and only one $\bar{a}$ with $0\leq \bar{a}\leq p-1$ such that $a\;\cdot \;\bar{a}\equiv 1\mathrm{mod}p$. Let *N*(*p*) denote the number of all 1 ≤ *a* ≤ *p* − 1 in which *a* and $\bar{a}$ are of opposite parity. Professor D. H. Lehmer [1] asked us to study *N*(*p*) or at least to say something nontrivial about it. It is known that *N*(*p*) ≡ 2 or  0mod⁡4 when *p* ≡ ±1mod⁡4. Some works related to the Lehmer problem can be found in references [2–5]. For example, Zhang [2, 4] proved the asymptotic formula

(1) $$\begin{array}{c} N\left(p\right)=\frac{1}{2}\cdot p+O\left(p^{1/2}\cdot \ln^{2}p\right). \end{array}$$

In this paper, we will study a new summation related to the Lehmer problem. For any fixed integer *k* ≥ 2 and integer *r* with (*r*, *p*) = 1, we define the sums *N*(*k*, *r*; *p*) as follows:

(2) $$\begin{array}{c} N\left(k,r;p\right)=\frac{1}{2}\underset{a_{1}a_{2}\cdots a_{k}\equiv r\mathrm{mod}p}{\overset{p-1}{\underset{a_{1}=1}{\sum }}\;\overset{p-1}{\underset{a_{2}=1}{\sum }}\cdots \overset{p-1}{\underset{a_{k}=1}{\sum }}}\left(1-{\left(-1\right)}^{a_{1}+a_{2}+\cdots +a_{k}}\right). \end{array}$$

In fact, *N*(*k*, *r*; *p*) is a generalization of the Lehmer problem. For example, if *k* = 2 and *r* = 1, then from the definition of *N*(2,1; *p*) we have

(3) N(2,1;p)=12∑a=1p−1∑b=1p−1ab≡1mod⁡p(1−(−1)a+b)=12∑a=1p−1(1−(−1)a+a¯)=N(p).

So *N*(2,1; *p*) becomes *N*(*p*), the Lehmer problem.

Now we are concerned about the arithmetical properties of *N*(*k*, *r*; *p*). This problem is interesting, because it is a generalization of the Lehmer problem.

In this paper, we use the analytic method and the estimate for high-dimension Kloosterman sums to study the asymptotic properties of *N*(*k*, *r*; *p*) and give two interesting asymptotic formulae for it. That is, we will prove the following.

**Theorem 1 .**

Let *p* be an odd prime. Then for any fixed integer *k* ≥ 2 and integer *r* with (*r*, *p*) = 1, we have the asymptotic formula

(4) $$\begin{array}{c} N\left(k,r;p\right)=\frac{1}{2}\cdot {\left(p-1\right)}^{k-1}+O\left(p^{\left(k-1\right)/2}\cdot \ln^{k}p\right). \end{array}$$

In order to facilitate the description of Theorem 2, we need to give the definition of high-dimension Kloosterman sums *C*(*k*, *m*; *q*). Let *q* ≥ 3 be an integer. For any integer *m*, we define

(5) C(k,m;q) =∑a1=1q(a1,q)=1 ∑a2=1q(a2,q)=1⋯  ∑ak=1q(ak,q)=1e(a1+a2+⋯+ak+ma¯1a¯2⋯a¯kq),

where $e(y)=e^{2\pi iy},a_{i}\cdot {\bar{a}}_{i}\equiv 1\mathrm{mod}p,i=1,2,\ldots ,k$.

About some arithmetical properties of *C*(*k*, *m*; *q*), one can find them in [6–8]. Let *E*(*k*, *r*; *p*) = *N*(*k*, *r*; *p*)−(1/2)(*p* − 1)^*k*−1^ denote the error term in the asymptotic formula of *N*(*k*, *r*; *p*). As another main content of this paper, we will study the asymptotic properties of the hybrid mean value of $C(k-1,r{\bar{2}}^{k};p)$ and *E*(*k*, *r*; *p*) and also give a sharp asymptotic formula for it. That is, we will prove the following.

**Theorem 2 .**

Let *p* be an odd prime. Then for any fixed integer *k* ≥ 2, we have the asymptotic formula

(6) ∑r=1p−1C(k−1,r2¯k;p)·E(k,r;p) =−4k−1·ikπk·pk+O(pk−1+ϵ),

where *i* ^2^ = −1, *ϵ* denotes any fixed positive number.

The constants ${\bar{2}}^{k}$ in Theorem 2 cannot be omitted. Otherwise, the main term in Theorem 2 is zero. If *k* = 3 and 4, then from Theorem 2 we can also deduce the following two corollaries.

**Corollary 3 .**

Let *p* be an odd prime. Then for any fixed positive number *ϵ* > 0, we have the asymptotic formula

(7) ∑r=1p−1C(2,8¯r;p)·E(3,r;p)=16·iπ3·p3+O(p2+ϵ).

**Corollary 4 .**

Let *p* be an odd prime. Then for any fixed positive number *ϵ* > 0, we have the asymptotic formula

(8) ∑r=1p−1C(3,16¯r;p)·E(4,r;p)=−64π4·p4+O(p3+ϵ).

## 2. Several Lemmas

In this section, we will give several lemmas, which are necessary in the proofs of our theorems. Hereinafter, we will use many properties of Gauss sums and the estimate for high-dimension Kloosterman sums; all of these contents can be found in references [6, 9], so they will not be repeated here. First we have the following.

**Lemma 5 .**

Let *p* be an odd prime. Then for fixed integer *k* ≥ 1 and any integer *m*, we have the estimate

(9) |C(k,m;p)| =|∑a1=1p−1 ∑a2=1p−1⋯∑ak=1p−1e(a1+a2+⋯+ak+ma¯1a¯2⋯a¯kp)| ≪pk/2.

**Proof**

See [6, 7].

**Lemma 6 .**

Let *p* be an odd prime. Then for any odd character *χ*mod⁡*p* (i.e., *χ*(−1) = −1), we have the identity

(10) $$\begin{array}{c} \overset{p-1}{\underset{a=1}{\sum }}{\left(-1\right)}^{a}\cdot \chi \left(a\right)\;=2\left(1-2\chi \left(2\right)\right)\cdot \left(\frac{1}{p}\cdot \overset{p-1}{\underset{a=1}{\sum }}a\cdot \chi \left(a\right)\right). \end{array}$$

**Proof**

See [10] or Lemma  3 in [3].

**Lemma 7 .**

Let *p* be an odd prime. Then for any integer (*r*, *p*) = 1, we have

(11) N(k,r;p) =12·(p−1)k−1−2k−1·ikπk(p−1)  ×∑χ(−1)=−1χ¯(r)(1−2χ(2))kτk(χ)Lk(1,χ¯),

where ∑_*χ*(−1)=−1_ denotes the summation over all odd characters *χ*mod⁡*p*, *τ*(*χ*) = ∑_*a*=1_^*p*−1^ *χ*(*a*) · *e*(*a*/*p*) denotes the classical Gauss sums, and *L*(*s*, *χ*) denotes the Dirichlet *L*-function corresponding to *χ*mod⁡*p*.

**Proof**

From the orthogonality of characters mod⁡ *p* and the definition of *N*(*k*, *r*; *p*) we have the identity

(12) N(k,r;p) =12∑a1=1p−1 ∑a2=1p−1⋯∑ak=1p−1a1a2⋯ak≡rmod⁡p(1−(−1)a1+a2+⋯+ak) =12·(p−1)k−1−12∑a1=1p−1 ∑a2=1p−1⋯∑ak=1p−1a1a2⋯ak≡rmod⁡p(−1)a1+a2+⋯+ak =12·(p−1)k−1−12(p−1)  ×∑χmod⁡pχ¯(r)(∑a=1p−1(−1)a·χ(a))k ≡12·(p−1)k−1−A(k,p).

For any odd character *χ*mod⁡*p*, from Theorems 12.11 and 12.20 of [9] we have

(13) $$\begin{array}{c} \frac{1}{p}\cdot \overset{p-1}{\underset{a=1}{\sum }}a\cdot \chi \left(a\right)=\frac{i}{\pi }\cdot \tau \left(\chi \right)\cdot L\left(1,\bar{\chi }\right). \end{array}$$

Note that, for any even character *χ*mod⁡*p*, we have the identity

(14) $$\begin{array}{c} \overset{p-1}{\underset{a=1}{\sum }}{\left(-1\right)}^{a}\cdot \chi \left(a\right)=0, \end{array}$$

from (13) and Lemma 6 we have

(15) A(k,p)=2k−1·ikπk(p−1) ×∑χ(−1)=−1χ¯(r)(1−2χ(2))kτk(χ)Lk(1,χ¯).

Now Lemma 7 follows from (12) and (15).

**Lemma 8 .**

Let *p* be an odd prime and *k* a fixed integer with *k* ≥ 2. Then for any nonprincipal character *χ*mod⁡*p* and any real numbers *y* ≥ *p* ^3^, we have the estimate

(16) $$\begin{array}{c} \left|\underset{n\leq y}{\sum }\chi \left(n\right)\cdot d_{k}\left(n\right)\right|\ll y^{1-(1/2^{k-1})}\cdot \sqrt{p}\cdot \ln p. \end{array}$$

**Proof**

We use mathematical induction to prove this lemma. If *k* = 2, then from the Pòlya-Vinogradov inequality we have

(17) |∑n≤yχ(n)d(n)| =|∑mn≤yχ(mn)| =|2∑n≤yχ(n)∑m≤y/nχ(m)−(∑n≤yχ(n))2| ≪ypln⁡p=y1−(1/2)·p·ln⁡p.

Assume that the lemma holds for *k* = *r*. That is,

(18) $$\begin{array}{c} \left|\underset{n\leq y}{\sum }\chi \left(n\right)\cdot d_{r}\left(n\right)\right|\ll y^{1-(1/2^{r-1})}\cdot \sqrt{p}\cdot \ln p. \end{array}$$

Then for *k* = *r* + 1, note that *d*_*r*+1_(*n*) = ∑_*s*∣*n*_ *d*_*r*_(*s*); applying estimate (18) and the Pòlya-Vinogradov inequality we have

(19) |∑n≤yχ(n)dr+1(n)| =|∑mn≤yχ(mn)dr(m)| ≤|∑n≤yχ(n)∑m≤y/nχ(m)dr(m)  +∑m≤yχ(m)dr(m)∑n≤y/nχ(n)|  +|∑n≤yχ(n)|·|∑m≤yχ(m)dr(m)| ≪∑n≤y(yn)1−(1/2r−1)·p·ln⁡p+y1−(1/2r)·p·ln⁡p ≪y1−(1/2r)·p·ln⁡p.

Now our lemma follows from the induction.

## 3. Proofs of the Theorems

In this section, we will prove our conclusions. First we prove Theorem 1. For any real number *N* ≥ *p* ^*k*^, applying Abel's identity (see Theorem 4.2 of [9]) we have

(20) Lk(1,χ¯)=∑1≤n≤Nχ¯(n)dk(n)n +∫N∞1y2(∑N<n≤yχ¯(n)dk(n))dy.

For any integer 0 ≤ *i* ≤ *k*, from Lemma 5 and the definition of *C*(*k*, *m*; *p*) we have

(21) ∑χ(−1)=−1χ¯(r)χ(2i)τk(χ)∑1≤n≤Nχ¯(n)dk(n)n =p−12∑1≤n≤N(n,p)=1dk(n)n  ×∑a1=1p−1⋯∑ak−1=1p−1e(a1+⋯+ak−1+nr2i¯·a¯1a¯2⋯a¯k−1p)  −p−12∑1≤n≤N(n,p)=1dk(n)n  ×∑a1=1p−1⋯∑ak−1=1p−1e(a1+⋯+ak−1−nr2i¯·a¯1a¯2⋯a¯k−1p) ≪p−12∑1≤n≤N(n,p)=1dk(n)n·p(k−1)/2≪p(k+1)/2·ln⁡kN.

Applying (20) and the binomial expression we have the estimate

(22) ∑χ(−1)=−1χ¯(r)(1−2χ(2))kτk(χ)∑1≤n≤Nχ¯(n)dk(n)n ≪p(k+1)/2·ln⁡kN.

Taking *N* = *p* ^2^*k*−1^^, note that $|\tau (\chi )|=\sqrt{p}$ and the identity

(23) $$\begin{array}{c} \underset{N<n\leq y}{\sum }\bar{\chi }\left(n\right)d_{k}\left(n\right)=\underset{n\leq y}{\sum }\bar{\chi }\left(n\right)d_{k}\left(n\right)-\underset{n\leq N}{\sum }\bar{\chi }\left(n\right)d_{k}\left(n\right), \end{array}$$

and applying Lemma 8 we have the estimate

(24) |∑χ(−1)=−1χ¯(r)(1−2χ(2))kτk(χ)×∫N∞1y2(∑N<n≤yχ¯(n)dk(n))dy| ≪p(k+2)/2·pN1/2k−1·ln⁡p=p(k+1)/2·ln⁡p.

Combining (20), (22), (24), and Lemma 7 we may immediately deduce the asymptotic formula

(25) $$\begin{array}{c} N\left(k,r;p\right)=\frac{1}{2}\cdot {\left(p-1\right)}^{k-1}+O\left(p^{\left(k-1\right)/2}\cdot \ln^{k}p\right). \end{array}$$

The proof of Theorem 1 is right.

Now we prove Theorem 2. For any nonprincipal character *χ*mod⁡*p*, from the definition and properties of Gauss sums we have

(26) ∑r=1p−1χ¯(r)·C(k−1,r2¯k;p) =∑r=1p−1χ¯(r)  ×∑a1=1p−1∑a2=1p−1⋯∑ak−1=1p−1e(a1+a2+⋯+ak−1+r2¯ka¯1a¯2⋯a¯k−1p) =∑a1=1p−1⋯∑ak−1=1p−1e(a1+a2+⋯+ak−1p)  ×∑r=1p−1χ¯(r)e(r2¯ka¯1a¯2⋯a¯k−1p) =τ(χ¯)χ¯(2k)  ×∑a1=1p−1⋯∑ak−1=1p−1χ(a¯1a¯2⋯a¯k−1)e(a1+a2+⋯+ak−1p) =χ¯(2k)·τk(χ¯).

Note that $\tau^{k}\left(\bar{\chi }\right)\cdot \tau^{k}(\chi )={\bar{\chi }}^{k}(-1){\bar{\tau (\chi )}}^{k}\cdot \tau^{k}(\chi )={\bar{\chi }}^{k}(-1)\cdot p^{k}$; from Lemma 7 and the definition of *E*(*k*, *r*; *p*) we have

(27) ∑r=1p−1C(k−1,r2¯k;p)·E(k,r;p) =−2k−1·ikπk(p−1)·pk  ·∑χ(−1)=−1(−1)k(1−2χ(2))kχ¯(2k)Lk(1,χ¯) =(−1)k+12k−1·ikπk(p−1)·pk  ·∑j=0k(jk)(−1)j·2j·∑χ(−1)=−1χ¯(2k−j)∑n=1∞χ¯(n)·dk(n)n =(−1)k+12k−1·ikπk(p−1)·pk·p−12(−1)k·2k+O(pk−1+ϵ) =−4k−1·ikπk·pk+O(pk−1+ϵ),

where *i* ^2^ = −1, $\left(\begin{array}{c} j\\ k \end{array}\right)=k!/\left(j!\cdot \left(k-j\right)!\right)$, *ϵ* denotes any fixed positive number and *d*_*k*_(*n*) denotes the *k*th divisor function. That is, *d*_*k*_(*n*) = (∑_*d*_1_∣*n*_∑_*d*_2_∣*n*_⋯∑_*d*_*k*_∣*n*_)_*d*_1_*d*_2_⋯*d*_*k*_=*n*_1.

The proof of Theorem 2 is right.
